# Cytokine Release Syndrome (CRS) and Nicotine in COVID-19 Patients: Trying to Calm the Storm

**DOI:** 10.3389/fimmu.2020.01359

**Published:** 2020-06-11

**Authors:** Jesus Gonzalez-Rubio, Carmen Navarro-Lopez, Elena Lopez-Najera, Ana Lopez-Najera, Lydia Jimenez-Diaz, Juan D. Navarro-Lopez, Alberto Najera

**Affiliations:** ^1^Centre for Biomedical Research, School of Medicine, University of Castilla-La Mancha, Albacete, Spain; ^2^Hospital General La Mancha Centro, Servicio de Salud de Castilla-La Mancha, Ciudad Real, Spain; ^3^Gerencia de Atención Primaria, Salud de Castilla y Leon, Avila, Spain; ^4^Gerencia de Emergencias Sanitarias, Salud de Castilla y Leon, Spain; ^5^Centre for Biomedical Research, School of Medicine, University of Castilla-La Mancha, Ciudad Real, Spain

**Keywords:** cholinergic anti-inflammatory pathway, nicotine, Cytokine Release Syndrom (CRS), SARS-CoV-2 (virus), COVID- 19, lung

## Abstract

SARS-CoV-2 is a new coronavirus that has caused a worldwide pandemic. It causes severe acute respiratory syndrome (COVID-19), which is fatal in many cases, and is characterized by a cytokine release syndrome (CRS). Great efforts are currently being made to block the signal transduction pathway of pro-inflammatory cytokines in order to control this “cytokine storm” and rescue severely affected patients. Consequently, possible treatments for cytokine-mediated hyperinflammation, preferably within approved safe therapies, are urgently being researched to reduce rising mortality. One approach to inhibit proinflammatory cytokine release is to activate the cholinergic anti-inflammatory pathway through nicotinic acetylcholine receptors (α7nAchR). Nicotine, an exogenous α7nAchR agonist, is clinically used in ulcerative colitis to counteract inflammation. We have found epidemiological evidence, based on recent clinical SARS-CoV-2 studies in China, that suggest that smokers are statistically less likely to be hospitalized. In conclusion, our hypothesis proposes that nicotine could constitute a novel potential CRS therapy in severe SARS-CoV-2 patients.

## Introduction

SARS-CoV-2 is a new coronavirus that originated in Wuhan (Hubei Province, China) in December 2019, and it has already developed into a pandemic with worldwide spread (1). It causes a severe acute respiratory syndrome (2). SARS-CoV-2 is the third coronavirus outbreak to occur this current century, following severe acute respiratory syndrome coronavirus (SARS-CoV) and Middle East respiratory syndrome coronavirus (MERS-CoV) (3).

SARS-CoV-2 causes varying degrees of illness degrees. Fever and cough are dominant symptoms, but severe disease also occurs. Then, when COVID-19 patients' aggravation is presented, lung hyperinflammation may occur due to a virus-activated “cytokine storm” or cytokine release syndrome (CRS) (4). Among different cytokines increased in such exacerbated response (5), Interleukin-6 (IL-6) in serum is mainly expected to predict the severity of SARS-CoV-2 pneumonia, as suppression of pro-inflammatory IL-6 have been shown to have a therapeutic effect in many inflammatory diseases, including viral infections (6).

In severe cases, SARS-CoV-2 has been shown to activate both the innate and adaptive immune system in the alveolar tissue, inducing the release of many cytokines and subsequent cytokine release syndrome (7). During this response, levels of pro-inflammatory cytokines (including TNFa, interleukin (IL)-1b, IL-6, and IL-8) are increased (5), which is also an important cause of death (8). Therefore, one may think that controlling such crucial inflammatory factors could be a successful approach to reduce mortality in severe patients.

## Could the Cytokine Storm be Controlled by a Physiological Protective Anti-Inflammatory Mechanism?

The existence of a cholinergic anti-inflammatory pathway that modulates inflammatory responses during systemic inflammation has been demonstrated (9). a7-nicotinic acetylcholine receptor (a7nAChR) are known to be expressed on macrophages and to be essential for attenuating the inflammatory response by calcium influx-mediated activation during systemic inflammation (10). The underlying mechanism conveys that activation of a7nAChR on infiltrated inflammatory cells, including macrophages and neutrophils, induces suppression of NF-kB activation (11) and secretion of pro-inflammatory cytokines and chemokines from inflammatory cells, including alveolar macrophages (12). Interestingly, it has been already reported that nicotine, an a7nAChR agonist, exerts an anti-inflammatory effect in a murine model of acute lung injury (13). In fact, in other inflammatory diseases, such as ulcerative Colitis (UC), smoking or treatment with nicotine has demonstrated to significantly decrease the risk of developing the disease (14).

## The Nicotine-COVID19 Hypothesis

In this scenario, we hypothesize that nicotine could ameliorate the cytokine storm and severe related inflammatory response through a7nAChR-mediated cholinergic anti-inflammatory pathway. Nicotine is an accessible, existing, and approved treatment, with described side effects, that could likely reduce the rising mortality in the short term.

## Support for the Hypothesis

Paradoxically, it is well-stablished that smokers have a significantly increased risk of chronic respiratory disease and acute respiratory infections. Current smokers have a higher risk of developing influenza compared to non-smokers (15). Smoking is also significantly associated with MERS-CoV (16), and there is no clear evidence for SARS-CoV-2 (17).

In China, 54.0% of men are current smokers, whereas only 2.6% of women smoke (18); it should therefore be expected that the number of current smokers hospitalized with SARS-CoV-2 should be in a similar or larger percentage with male predominance. However, surprisingly, the number of hospitalized smoking patients in the Chinese outbreak is much lower than expected (19–23). In Table 1 we show results comparing, both separately and using a combined approach, proportions of hospitalized smokers with SARS-CoV-2 in five different studies. Combined total current smokers was 159 compared to the 526·0 expected considering the male/female current smokers' ratio in China. We performed a χ^2^-test or Fisher's exact test to compare differences between observed and expected current smoker, for all the studies individually and combining all data, and we found significant differences in all cases (*p* < 0·001). Similar data have been reported for the ongoing pandemic in Europe and America, from patients in Italy (24) and US (25), which strongly support our hypothesis.

**Table 1:** Comparison of hospitalized current smokers in the Chinese COVID19 outbreak.

**References**	***N* (male/female)**	**Median age (years)**	**Current smokers**	**Expected current smokers (male/female)**	**Sig**.
Zhou et al. (22)	191 (119, 72)	56.0 (IQR: 46.0–67.0)	11	66.2 (64.3, 1.9)	*p* < 0.0001
Guan et al. (19)	1096 (637, 459)	47.0 (IQR: 35.0–58.0)^*^	137	355.9 (344.0, 11.9)	*p* < 0.0001
Huang et al. (20)	41 (30, 11)	49.0 (IQR: 41.0–58.0)	3	16.5 (16.2, 0.3)	*p* = 0.0006
Zhang et al. (21)	140 (69, 71)	57.0 (Range: 25.0–87.0)	2	39.2 (37.3, 1.9)	*p* < 0.0001
Mo et al. (23)	155 (86, 69)	54.0 (IQR: 42.0–66.0)	6	48.2 (46.4, 1.8)	*p* < 0.0001
Combined	1623 (941, 682)		159	526.0 (508.2, 17.8)	*p* < 0.0001

* *0.9% of the patients were younger than 15 years of age*.

*The combined analysis is the result of the summation of all individual studies. Studies included in the analysis were selected on the bases of an homogeneous report of both clinical and epidemiological data. All studies contained detailed data about hospitalized current smokers. All patients were adults. Comparisons within age ranges of current smokers and non-smokers could not be performed. All patients had been diagnosed with COVID-19 by PCR tests. Data were taken from electronic medical records. IQR, interquartile range*.

On the other hand, there are different formulations for nicotine administration: gums, patches, inhalators, nasal and oral sprays, sublingual tablets, and electronic cigarettes. Apart from the electronic cigarettes, which are quite new, the previous options are considered relatively safe, as most side effects are mild (26). For instance, in the clinical practice transdermal nicotine is administered at high tolerated dosage for controlling clinical manifestations of chronic UC. For acute SARS-CoV-2 treatment to ameliorate hyperinflammation, nicotine dosage and pharmaceutical form could be chosen according to previous experience with UC (14, 27). In addition to nicotine, it has been suggested that both selective α7nAChR agonists and allosteric modulators may be potential tools for the treatment of acute lung injury (12, 28, 29).

To our knowledge, no clinical trials of nicotine in COVID19 patients are currently being run. The most promising trial under run is the one using Tocilizumab, a blocker of IL-6 receptor for the treatment of cytokine storm (6), so it is expected to be an effective drug of severe patients.

To further complete present hypothesis, factors such as age, smoking habits (*vg*. nicotine administration route), or any others that could influence smoker's health and susceptibility to the infection should be considered in future studies.

## Conclusions

It has been observed that the number of current smokers hospitalized in the SARS-CoV-2 outbreak in China is lower than expected when compared to the prevalence of smoking in this country. It has been described that, due to a cytokine storm, many patients are aggravated, showing severe inflation in the lungs. Our proposal may be easily tested in the short term, as it takes advantage of the existence of a cholinergic anti-inflammatory pathway that physiologically modulates inflammatory responses during systemic inflammation effect. Controlling the cytokine storm using nicotinic administration could then be expected to become a new method for the treatment of severe patients, as it has already been proved in UC patients.

Just very recently, a group of recognized experts in the field recommended the “identification and treatment of hyperinflammation using existing, approved therapies with proven safety profiles to address the immediate need to reduce the rising mortality” (8). Current treatment with Tocilizumab seems to be useful to control cytokines storm. However, very strict criteria for its clinical use limits its availability, mainly due to price and adverse effects. Our proposal, recently supported by different non peer-reviewed additional reports (30, 31), suggest that the treatment with nicotine by using any of the already approved pharmaceutical forms available in the market could reduce the lung inflammatory response by controlling the cytokines storm and, therefore, the number of patients in need of hospitalization for aggravation in the SARS-CoV-2 outbreak.

## Data Availability Statement

The raw data supporting the conclusions of this article will be made available by the authors, without undue reservation.

## Author Contributions

JG-R and AN designed the study and collected data. JG-R, LJ-D, JN-L, and AN wrote the manuscript. All authors analyzed and interpreted the data. All authors contributed to the article and approved the submitted version.

## Conflict of Interest

The authors declare that the research was conducted in the absence of any commercial or financial relationships that could be construed as a potential conflict of interest.
